# 6-[(*E*)-3,7-Dimethyl­octa-2,6-dien­yl]-5,7-dihydr­oxy-8-(2-methyl­butano­yl)-4-phenyl-2*H*-chromen-2-one from *Mesua kunstleri* King (Kosterm)

**DOI:** 10.1107/S1600536808018151

**Published:** 2008-06-25

**Authors:** Gomathi Chan, Khalijah Awang, A. Hamid A. Hadi, Seik Weng Ng

**Affiliations:** a Department of Chemistry, University of Malaya, 50603 Kuala Lumpur, Malaysia

## Abstract

The title compound, C_30_H_34_O_5_, crystallizes with two symmetry-independent mol­ecules in the asymmetric unit. In the crystal structure, the two independent mol­ecules are disposed about a pseudo-center of inversion. An intra­molecular O—H⋯O hydrogen bond is observed in each independent mol­ecule. The crystal structure is stabilized by C—H⋯O hydrogen bonds.

## Related literature

For the spectroscopic analysis of the title compound, see: Verotta *et al.* (2004 ▶).
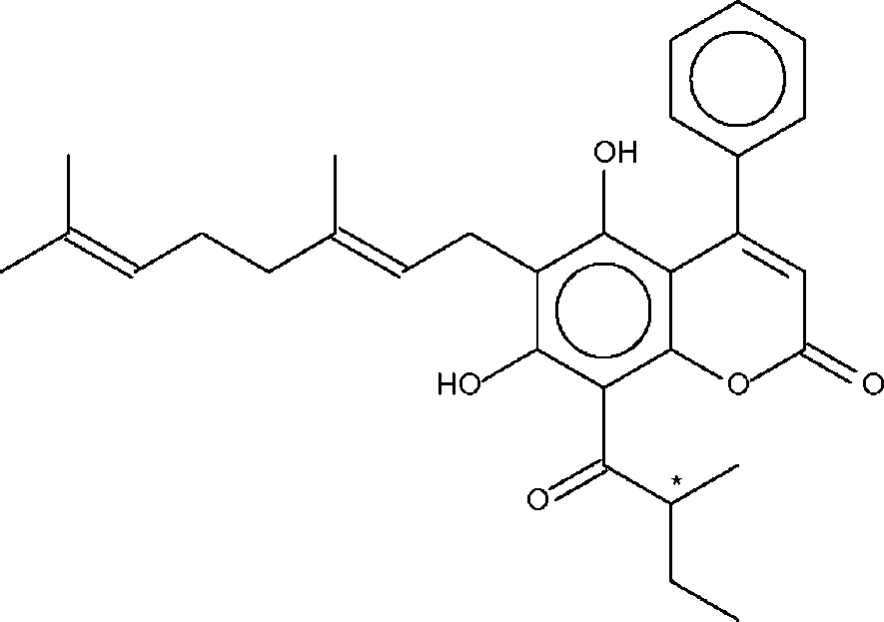


## Experimental

### 

#### Crystal data

C_30_H_34_O_5_
*M* *_r_* = 474.57
Triclinic, 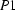

*a* = 5.6729 (1) Å
*b* = 13.4350 (3) Å
*c* = 16.8404 (3) Å
α = 87.550 (1)°
β = 82.243 (1)°
γ = 85.332 (1)°
*V* = 1266.88 (4) Å^3^
*Z* = 2
Mo *K*α radiation
μ = 0.08 mm^−1^
*T* = 100 (2) K
0.40 × 0.08 × 0.04 mm

#### Data collection

Bruker SMART APEX diffractometer
Absorption correction: none
16336 measured reflections
5783 independent reflections
4459 reflections with *I* > 2σ(*I*)
*R*_int_ = 0.034

#### Refinement

*R*[*F* ^2^ > 2σ(*F* ^2^)] = 0.070
*wR*(*F* ^2^) = 0.210
*S* = 1.04
5783 reflections
645 parameters
65 restraints
H-atom parameters constrained
Δρ_max_ = 1.21 e Å^−3^
Δρ_min_ = −0.44 e Å^−3^

### 

Data collection: *APEX2* (Bruker, 2007 ▶); cell refinement: *SAINT* (Bruker, 2007 ▶); data reduction: *SAINT*; program(s) used to solve structure: *SHELXS97* (Sheldrick, 2008 ▶); program(s) used to refine structure: *SHELXL97* (Sheldrick, 2008 ▶); molecular graphics: *X-SEED* (Barbour, 2001 ▶); software used to prepare material for publication: *publCIF* (Westrip, 2008 ▶).

## Supplementary Material

Crystal structure: contains datablocks I, global. DOI: 10.1107/S1600536808018151/ci2600sup1.cif

Structure factors: contains datablocks I. DOI: 10.1107/S1600536808018151/ci2600Isup2.hkl

Additional supplementary materials:  crystallographic information; 3D view; checkCIF report

## Figures and Tables

**Table 1:** Hydrogen-bond geometry (Å, °)

*D*—H⋯*A*	*D*—H	H⋯*A*	*D*⋯*A*	*D*—H⋯*A*
O4—H4*o*⋯O5	0.84	1.70	2.438 (8)	145
O9—H9*o*⋯O10	0.84	1.71	2.452 (9)	147
C29—H29*b*⋯O5^i^	0.99	2.29	3.115 (10)	140
C32—H32⋯O2^ii^	0.95	2.43	3.347 (8)	161
C35—H35⋯O2^iii^	0.95	2.47	3.242 (9)	138
C39—H39⋯O8^iv^	0.95	2.55	3.434 (8)	155

Symmetry codes: (i) 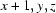; (ii) 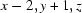; (iii) 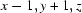; (iv) 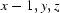.

**Fig. 1.**
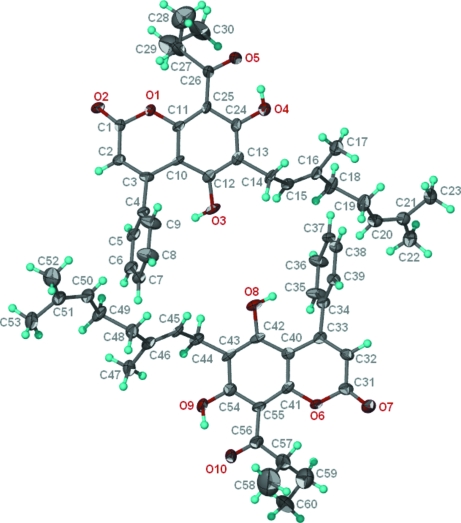
An ORTEP plot of the title compound, showing 70% probability displacement ellipsoids and the atomic numbering scheme. Hydrogen atoms are drawn as spheres of arbitrary radii. The two independent molecules are disposed over a false center of inversion.

## supplementary crystallographic information

### Comment

The title compound, previously isolated from *Mesua ferrea*, has been evaluated as a multidrug-resistant antibacterial. The structure was elucidated from spectroscopic measurements (Verotta *et al.*, 2004). Locally, the bark of *Mesua kunstleri *(King) Kosterm is used for the treatment of dyspepsis and renal diseases. The identical compound is isloated from the bark of this plant; the compound features the common coumarin skeleton, and is an 8-acyl-6-prenyl derivative. The spectroscopic assignment is now confirmed in the crystal structure analysis.

The title compound contains two independent molecules that are disposed over a false center of inversion (Fig. 1). An intramolecular O—H···O hydrogen bond is observed in each independent molecule. The crystal structure is stabilized by C—H···O hydrogen bonds (Table 1).

### Experimental

One-and-a-half kg of the bark of *Mesua kunstleri* King (Kosterm), (Clusiaceae), collected at Hutan Simpanan Rimba Teloi, Kedah, Malaysia [voucher specimen number KL4485], were extracted by maceration in hexane (6 *L*) for 72 h to yield a gummy residue (103 g). A portion (10 g) was column chromatographed over silica gel (300 g, 40–63 µm) by using hexane-ethyl acetate as eluents to give six fractions. The first fraction (6 g) was further chromatographed over silica gel (180 g, 5–40 µm) by using hexane:ethyl acetate (95:5). The title compound (1.5 g) was eluted with hexane:ethyl acetate (96:4).

### Refinement

For the 2-methylbutanoyl fragments, 1,2-related C–C distances were restrained to 1.54 (1) Å and 1,3-related ones to 2.51 (1) Å; the anisotropic displacement parameters of the carbon atoms (except the double-bond ones) were restrained to be nearly isotropic. H-atoms were placed in calculated positions (O—H = 0.84 Å and C—H = 0.95–0.99 Å) and were included in the refinement in the riding-model approximation, with *U*(H) set to 1.2–1.5*U*_eq_(C,*O*). The torsion angles of the hydroxy groups were refined. The final difference Fourier map had a large peak in the vicinity of the 2-methylbutanoyl fragment of a independent molecule. The chirality of the compound is assumed from the reported optical activity measurements (Verotta *et al.*, 2004).

### Crystal data

**Table:**

C_30_H_34_O_5_	*Z* = 2
*M**_r_* = 474.57	*F*_000_ = 508
Triclinic, *P*1	*D*_x_ = 1.244 Mg m^−^^3^
Hall symbol: P 1	Mo *K*α radiation λ = 0.71073 Å
*a* = 5.6729 (1) Å	Cell parameters from 3286 reflections
*b* = 13.4350 (3) Å	θ = 2.4–28.2º
*c* = 16.8404 (3) Å	µ = 0.08 mm^−^^1^
α = 87.550 (1)º	*T* = 100 (2) K
β = 82.243 (1)º	Prism, colourless
γ = 85.332 (1)º	0.40 × 0.08 × 0.04 mm
*V* = 1266.88 (4) Å^3^	

### Data collection

**Table:**

Bruker SMART APEX diffractometer	4459 reflections with *I* > 2σ(*I*)
Radiation source: fine-focus sealed tube	*R*_int_ = 0.034
Monochromator: graphite	θ_max_ = 27.5º
*T* = 100(2) K	θ_min_ = 1.2º
ω scans	*h* = −7→7
Absorption correction: None	*k* = −17→13
16336 measured reflections	*l* = −21→21
5783 independent reflections	

### Refinement

**Table:**

Refinement on *F*^2^	Secondary atom site location: difference Fourier map
Least-squares matrix: full	Hydrogen site location: inferred from neighbouring sites
*R*[*F*^2^ > 2σ(*F*^2^)] = 0.070	H-atom parameters constrained
*wR*(*F*^2^) = 0.210	*w* = 1/[σ^2^(*F*_o_^2^) + (0.111*P*)^2^ + 1.3027*P*] where *P* = (*F*_o_^2^ + 2*F*_c_^2^)/3
*S* = 1.04	(Δ/σ)_max_ = 0.001
5783 reflections	Δρ_max_ = 1.21 e Å^−^^3^
645 parameters	Δρ_min_ = −0.44 e Å^−^^3^
65 restraints	Extinction correction: none
Primary atom site location: structure-invariant direct methods	

### Special details

**Table:**

Geometry. All e.s.d.'s (except the e.s.d. in the dihedral angle between two l.s. planes) are estimated using the full covariance matrix. The cell e.s.d.'s are taken into account individually in the estimation of e.s.d.'s in distances, angles and torsion angles; correlations between e.s.d.'s in cell parameters are only used when they are defined by crystal symmetry. An approximate (isotropic) treatment of cell e.s.d.'s is used for estimating e.s.d.'s involving l.s. planes.

### Fractional atomic coordinates and isotropic or equivalent isotropic displacement parameters (Å^2^)

**Table:**

	*x*	*y*	*z*	*U*_iso_*/*U*_eq_	
O1	0.5000 (7)	0.5000 (3)	0.5000 (2)	0.0199 (9)	
O2	0.8130 (8)	0.4223 (3)	0.5477 (3)	0.0251 (10)	
O3	0.2134 (7)	0.8422 (3)	0.5466 (3)	0.0248 (10)	
H3O	0.3356	0.8419	0.5698	0.037*	
O4	−0.1436 (8)	0.6584 (4)	0.3762 (3)	0.0291 (10)	
H4O	−0.1506	0.5999	0.3614	0.044*	
O5	0.0088 (10)	0.4887 (4)	0.3416 (4)	0.0476 (15)	
O6	−0.5997 (7)	1.4425 (3)	0.7678 (3)	0.0224 (9)	
O7	−0.9094 (8)	1.5196 (4)	0.7193 (3)	0.0327 (12)	
O8	−0.2864 (8)	1.1053 (3)	0.7113 (3)	0.0260 (10)	
H8O	−0.3574	1.1133	0.6705	0.039*	
O9	0.0561 (8)	1.2875 (4)	0.8869 (3)	0.0291 (11)	
H9O	0.0288	1.3378	0.9156	0.044*	
O10	−0.1233 (12)	1.4518 (5)	0.9302 (4)	0.060 (2)	
C1	0.6762 (10)	0.4958 (5)	0.5509 (3)	0.0185 (12)	
C2	0.6655 (10)	0.5786 (5)	0.6033 (4)	0.0202 (12)	
H2	0.7705	0.5766	0.6429	0.024*	
C3	0.5081 (10)	0.6595 (5)	0.5972 (4)	0.0207 (13)	
C4	0.4912 (10)	0.7375 (5)	0.6583 (4)	0.0191 (12)	
C5	0.6800 (10)	0.7969 (5)	0.6608 (4)	0.0227 (13)	
H5	0.8215	0.7867	0.6240	0.027*	
C6	0.6627 (11)	0.8711 (5)	0.7168 (4)	0.0253 (14)	
H6	0.7924	0.9111	0.7185	0.030*	
C7	0.4530 (11)	0.8868 (5)	0.7710 (4)	0.0278 (14)	
H7	0.4386	0.9390	0.8081	0.033*	
C8	0.2727 (12)	0.8279 (7)	0.7702 (5)	0.0399 (19)	
H8	0.1341	0.8366	0.8085	0.048*	
C9	0.2875 (12)	0.7538 (7)	0.7133 (5)	0.0380 (18)	
H9	0.1566	0.7142	0.7123	0.046*	
C10	0.3524 (10)	0.6652 (4)	0.5356 (4)	0.0180 (12)	
C11	0.3452 (10)	0.5805 (4)	0.4900 (3)	0.0180 (12)	
C12	0.2028 (10)	0.7510 (5)	0.5160 (4)	0.0202 (13)	
C13	0.0372 (10)	0.7467 (5)	0.4626 (4)	0.0203 (12)	
C14	−0.1347 (10)	0.8390 (5)	0.4488 (4)	0.0216 (13)	
H14A	−0.2871	0.8165	0.4363	0.026*	
H14B	−0.1691	0.8769	0.4987	0.026*	
C15	−0.0330 (10)	0.9071 (5)	0.3812 (4)	0.0200 (12)	
H15	0.1083	0.9370	0.3886	0.024*	
C16	−0.1191 (11)	0.9300 (5)	0.3123 (4)	0.0233 (13)	
C17	−0.3265 (12)	0.8834 (5)	0.2859 (5)	0.0329 (16)	
H17A	−0.3609	0.8235	0.3194	0.049*	
H17B	−0.2869	0.8650	0.2297	0.049*	
H17C	−0.4669	0.9314	0.2914	0.049*	
C18	−0.0055 (11)	1.0067 (5)	0.2547 (4)	0.0286 (15)	
H18A	0.0526	0.9750	0.2030	0.034*	
H18B	0.1343	1.0293	0.2763	0.034*	
C19	−0.1767 (12)	1.0977 (6)	0.2398 (4)	0.0316 (15)	
H19A	−0.2999	1.0775	0.2087	0.038*	
H19B	−0.2584	1.1218	0.2920	0.038*	
C20	−0.0517 (12)	1.1810 (5)	0.1955 (4)	0.0292 (14)	
H20	0.0787	1.2020	0.2188	0.035*	
C21	−0.0959 (11)	1.2300 (5)	0.1281 (4)	0.0260 (13)	
C22	0.0533 (13)	1.3134 (5)	0.0928 (4)	0.0330 (15)	
H22A	0.1836	1.3193	0.1246	0.049*	
H22B	−0.0468	1.3764	0.0934	0.049*	
H22C	0.1197	1.2985	0.0374	0.049*	
C23	−0.2890 (12)	1.2083 (6)	0.0812 (4)	0.0300 (14)	
H23A	−0.3867	1.1584	0.1107	0.045*	
H23B	−0.2182	1.1824	0.0291	0.045*	
H23C	−0.3892	1.2698	0.0730	0.045*	
C24	0.0277 (10)	0.6593 (5)	0.4246 (3)	0.0195 (13)	
C25	0.1865 (11)	0.5725 (5)	0.4348 (4)	0.0215 (13)	
C26	0.1610 (13)	0.4843 (5)	0.3891 (5)	0.044 (2)	
C27	0.3025 (12)	0.3828 (5)	0.3980 (5)	0.072 (3)	
H27	0.3397	0.3771	0.4545	0.086*	
C28	0.148 (2)	0.2962 (6)	0.3877 (7)	0.074 (3)	
H28A	0.2373	0.2324	0.3982	0.111*	
H28B	0.1085	0.2984	0.3328	0.111*	
H28C	0.0013	0.3027	0.4256	0.111*	
C29	0.5363 (14)	0.3846 (7)	0.3452 (5)	0.075 (3)	
H29A	0.6287	0.3194	0.3500	0.089*	
H29B	0.6294	0.4368	0.3629	0.089*	
C30	0.4987 (19)	0.4062 (8)	0.2566 (5)	0.104 (4)	
H30A	0.6532	0.4003	0.2225	0.156*	
H30B	0.4240	0.4741	0.2507	0.156*	
H30C	0.3950	0.3580	0.2404	0.156*	
C31	−0.7703 (10)	1.4460 (5)	0.7166 (4)	0.0234 (13)	
C32	−0.7549 (10)	1.3652 (5)	0.6628 (4)	0.0238 (13)	
H32	−0.8627	1.3670	0.6241	0.029*	
C33	−0.5920 (10)	1.2867 (5)	0.6654 (4)	0.0201 (13)	
C34	−0.5701 (10)	1.2096 (5)	0.6026 (4)	0.0218 (13)	
C35	−0.3631 (11)	1.1987 (6)	0.5479 (5)	0.0331 (17)	
H35	−0.2382	1.2413	0.5494	0.040*	
C36	−0.3404 (11)	1.1258 (6)	0.4915 (5)	0.0353 (17)	
H36	−0.1975	1.1169	0.4551	0.042*	
C37	−0.5277 (11)	1.0645 (5)	0.4875 (4)	0.0267 (14)	
H37	−0.5129	1.0150	0.4481	0.032*	
C38	−0.7304 (11)	1.0768 (5)	0.5406 (4)	0.0261 (14)	
H38	−0.8567	1.0350	0.5387	0.031*	
C40	−0.4357 (10)	1.2785 (5)	0.7283 (3)	0.0194 (13)	
C41	−0.4404 (10)	1.3618 (5)	0.7759 (4)	0.0213 (13)	
C42	−0.2805 (10)	1.1951 (4)	0.7452 (4)	0.0191 (13)	
C39	−0.7539 (10)	1.1500 (5)	0.5975 (4)	0.0202 (12)	
H39	−0.8979	1.1592	0.6333	0.024*	
C43	−0.1161 (11)	1.1979 (5)	0.7973 (4)	0.0217 (13)	
C44	0.0599 (11)	1.1120 (5)	0.8118 (4)	0.0237 (13)	
H44A	0.2083	1.1388	0.8237	0.028*	
H44B	0.0998	1.0743	0.7619	0.028*	
C45	−0.0254 (10)	1.0410 (4)	0.8791 (4)	0.0202 (12)	
H45	−0.1601	1.0062	0.8721	0.024*	
C46	0.0662 (11)	1.0211 (5)	0.9474 (4)	0.0231 (13)	
C47	0.2795 (12)	1.0687 (6)	0.9704 (5)	0.0323 (15)	
H47A	0.3247	1.1221	0.9311	0.048*	
H47B	0.4137	1.0180	0.9712	0.048*	
H47C	0.2382	1.0968	1.0237	0.048*	
C48	−0.0360 (11)	0.9436 (5)	1.0062 (4)	0.0257 (14)	
H48A	−0.1772	0.9195	0.9865	0.031*	
H48B	−0.0910	0.9755	1.0581	0.031*	
C49	0.1398 (12)	0.8531 (5)	1.0205 (4)	0.0303 (15)	
H49A	0.2225	0.8299	0.9682	0.036*	
H49B	0.2617	0.8739	1.0520	0.036*	
C50	0.0163 (14)	0.7689 (5)	1.0643 (4)	0.0346 (16)	
H50	−0.1133	0.7469	1.0411	0.042*	
C51	0.0647 (12)	0.7206 (5)	1.1317 (4)	0.0292 (14)	
C52	−0.0778 (16)	0.6359 (7)	1.1650 (5)	0.047 (2)	
H52A	−0.1755	0.6169	1.1252	0.071*	
H52B	−0.1817	0.6566	1.2137	0.071*	
H52C	0.0304	0.5786	1.1779	0.071*	
C53	0.2580 (14)	0.7435 (6)	1.1803 (5)	0.0422 (19)	
H53A	0.3348	0.8027	1.1570	0.063*	
H53B	0.3771	0.6864	1.1798	0.063*	
H53C	0.1874	0.7562	1.2357	0.063*	
C54	−0.1128 (12)	1.2858 (5)	0.8400 (4)	0.0256 (14)	
C55	−0.2840 (12)	1.3680 (5)	0.8344 (4)	0.0264 (14)	
C56	−0.2909 (14)	1.4498 (5)	0.8911 (4)	0.045 (2)	
C57	−0.5006 (13)	1.5287 (5)	0.9073 (4)	0.059 (2)	
H57	−0.6360	1.5003	0.8858	0.071*	
C58	−0.583 (2)	1.5373 (11)	0.9978 (5)	0.090 (4)	
H58C	−0.7324	1.5796	1.0062	0.136*	
H58D	−0.4602	1.5670	1.0229	0.136*	
H58E	−0.6078	1.4706	1.0218	0.136*	
C59	−0.4514 (18)	1.6215 (6)	0.8552 (6)	0.082 (3)	
H59A	−0.5943	1.6694	0.8609	0.098*	
H59B	−0.4160	1.6033	0.7983	0.098*	
C60	−0.2386 (17)	1.6704 (7)	0.8803 (6)	0.075 (3)	
H60A	−0.2379	1.7394	0.8589	0.112*	
H60B	−0.0899	1.6327	0.8590	0.112*	
H60C	−0.2522	1.6702	0.9389	0.112*	

### Atomic displacement parameters (Å^2^)

**Table:**

	*U*^11^	*U*^22^	*U*^33^	*U*^12^	*U*^13^	*U*^23^
O1	0.025 (2)	0.015 (2)	0.0197 (19)	0.0034 (16)	−0.0062 (15)	−0.0007 (16)
O2	0.026 (2)	0.018 (2)	0.032 (2)	0.0049 (17)	−0.0069 (17)	−0.0085 (18)
O3	0.023 (2)	0.011 (2)	0.040 (3)	0.0011 (17)	−0.0022 (18)	−0.0050 (19)
O4	0.041 (2)	0.023 (3)	0.025 (2)	0.006 (2)	−0.0127 (19)	−0.003 (2)
O5	0.060 (3)	0.030 (3)	0.061 (3)	0.019 (2)	−0.043 (3)	−0.022 (3)
O6	0.025 (2)	0.014 (2)	0.029 (2)	0.0039 (16)	−0.0088 (17)	−0.0078 (17)
O7	0.022 (2)	0.029 (3)	0.048 (3)	0.0103 (19)	−0.0116 (19)	−0.016 (2)
O8	0.032 (2)	0.014 (2)	0.030 (2)	0.0008 (18)	−0.0002 (19)	−0.0053 (19)
O9	0.039 (3)	0.022 (3)	0.026 (2)	0.013 (2)	−0.0130 (19)	−0.003 (2)
O10	0.097 (5)	0.038 (3)	0.054 (3)	0.037 (3)	−0.058 (3)	−0.026 (3)
C1	0.019 (3)	0.017 (3)	0.019 (3)	0.001 (2)	−0.004 (2)	0.002 (2)
C2	0.016 (3)	0.018 (3)	0.026 (3)	0.002 (2)	−0.001 (2)	−0.006 (2)
C3	0.020 (3)	0.020 (3)	0.021 (3)	−0.002 (2)	0.001 (2)	−0.009 (2)
C4	0.017 (3)	0.015 (3)	0.025 (3)	0.001 (2)	−0.002 (2)	−0.005 (2)
C5	0.024 (3)	0.018 (3)	0.023 (3)	0.004 (2)	0.003 (2)	0.000 (2)
C6	0.024 (3)	0.018 (3)	0.034 (4)	0.001 (2)	−0.005 (2)	−0.001 (3)
C7	0.028 (3)	0.026 (4)	0.030 (3)	0.010 (3)	−0.008 (3)	−0.012 (3)
C8	0.027 (3)	0.050 (5)	0.041 (4)	−0.005 (3)	0.010 (3)	−0.024 (4)
C9	0.024 (3)	0.051 (5)	0.039 (4)	−0.007 (3)	0.005 (3)	−0.026 (4)
C10	0.021 (3)	0.008 (3)	0.024 (3)	0.000 (2)	0.001 (2)	−0.001 (2)
C11	0.025 (3)	0.012 (3)	0.016 (3)	0.003 (2)	0.001 (2)	0.000 (2)
C12	0.020 (3)	0.018 (3)	0.021 (3)	0.001 (2)	0.003 (2)	0.000 (2)
C13	0.021 (3)	0.020 (3)	0.019 (3)	0.002 (2)	0.002 (2)	0.002 (2)
C14	0.025 (3)	0.016 (3)	0.021 (3)	0.011 (2)	0.001 (2)	0.001 (2)
C15	0.019 (3)	0.019 (3)	0.021 (3)	0.003 (2)	0.001 (2)	−0.004 (2)
C16	0.027 (3)	0.018 (3)	0.024 (3)	0.008 (2)	−0.005 (2)	0.000 (2)
C17	0.031 (3)	0.026 (4)	0.043 (4)	0.001 (3)	−0.014 (3)	0.001 (3)
C18	0.030 (3)	0.026 (4)	0.025 (3)	0.010 (3)	0.004 (2)	0.007 (3)
C19	0.033 (3)	0.034 (4)	0.024 (3)	0.011 (3)	0.000 (2)	0.002 (3)
C20	0.036 (3)	0.027 (4)	0.025 (3)	0.002 (3)	−0.007 (2)	−0.007 (3)
C21	0.030 (3)	0.027 (3)	0.021 (3)	0.002 (2)	−0.003 (2)	−0.004 (2)
C22	0.042 (4)	0.028 (4)	0.029 (3)	−0.001 (3)	−0.009 (3)	0.007 (3)
C23	0.030 (3)	0.030 (4)	0.031 (3)	0.001 (3)	−0.008 (2)	0.003 (3)
C24	0.025 (3)	0.020 (3)	0.012 (3)	0.003 (2)	−0.002 (2)	0.005 (2)
C25	0.029 (3)	0.016 (3)	0.019 (3)	0.008 (2)	−0.008 (2)	0.000 (2)
C26	0.059 (4)	0.026 (4)	0.052 (4)	0.020 (3)	−0.040 (4)	−0.017 (3)
C27	0.077 (5)	0.062 (5)	0.087 (5)	0.045 (4)	−0.065 (5)	−0.053 (4)
C28	0.101 (7)	0.054 (5)	0.062 (5)	0.006 (5)	−0.002 (5)	0.002 (4)
C29	0.095 (6)	0.065 (5)	0.069 (5)	−0.007 (5)	−0.025 (5)	−0.014 (4)
C30	0.093 (6)	0.121 (7)	0.105 (6)	0.081 (5)	−0.064 (5)	−0.100 (6)
C31	0.017 (3)	0.023 (3)	0.030 (3)	−0.003 (2)	−0.002 (2)	−0.007 (3)
C32	0.020 (3)	0.025 (4)	0.028 (3)	−0.002 (2)	−0.006 (2)	−0.008 (3)
C33	0.016 (3)	0.017 (3)	0.027 (3)	−0.004 (2)	0.000 (2)	0.000 (2)
C34	0.022 (3)	0.019 (3)	0.024 (3)	0.000 (2)	−0.002 (2)	−0.004 (3)
C35	0.021 (3)	0.033 (4)	0.045 (4)	−0.010 (3)	0.006 (3)	−0.022 (3)
C36	0.017 (3)	0.043 (5)	0.046 (4)	0.000 (3)	0.001 (3)	−0.029 (3)
C37	0.029 (3)	0.018 (3)	0.036 (4)	0.003 (2)	−0.013 (3)	−0.011 (3)
C38	0.029 (3)	0.019 (3)	0.033 (3)	−0.005 (3)	−0.013 (3)	−0.002 (3)
C40	0.023 (3)	0.020 (3)	0.014 (3)	−0.003 (2)	0.001 (2)	0.000 (2)
C41	0.026 (3)	0.014 (3)	0.022 (3)	0.003 (2)	0.000 (2)	0.001 (2)
C42	0.023 (3)	0.010 (3)	0.021 (3)	−0.002 (2)	0.009 (2)	−0.001 (2)
C39	0.016 (3)	0.018 (3)	0.027 (3)	−0.008 (2)	−0.003 (2)	0.000 (2)
C43	0.029 (3)	0.011 (3)	0.021 (3)	0.004 (2)	0.007 (2)	0.002 (2)
C44	0.026 (3)	0.015 (3)	0.029 (3)	−0.001 (2)	0.001 (2)	0.000 (3)
C45	0.022 (3)	0.010 (3)	0.026 (3)	0.004 (2)	−0.001 (2)	0.001 (2)
C46	0.024 (3)	0.018 (3)	0.025 (3)	0.005 (2)	0.002 (2)	−0.003 (2)
C47	0.030 (3)	0.032 (4)	0.036 (4)	0.007 (3)	−0.013 (3)	−0.002 (3)
C48	0.030 (3)	0.026 (4)	0.019 (3)	0.003 (3)	−0.003 (2)	0.001 (2)
C49	0.041 (4)	0.017 (3)	0.030 (3)	0.006 (3)	−0.001 (3)	0.006 (3)
C50	0.057 (4)	0.021 (4)	0.027 (3)	0.003 (3)	−0.018 (3)	0.003 (3)
C51	0.045 (4)	0.021 (3)	0.021 (3)	0.004 (3)	−0.005 (2)	0.002 (2)
C52	0.065 (5)	0.043 (5)	0.036 (4)	−0.008 (4)	−0.013 (3)	0.010 (3)
C53	0.046 (4)	0.041 (5)	0.040 (4)	0.014 (3)	−0.018 (3)	0.000 (3)
C54	0.034 (3)	0.022 (4)	0.020 (3)	0.003 (3)	−0.004 (2)	−0.001 (3)
C55	0.045 (4)	0.013 (3)	0.021 (3)	0.004 (3)	−0.007 (3)	−0.004 (2)
C56	0.075 (5)	0.029 (4)	0.032 (3)	0.022 (4)	−0.026 (3)	−0.015 (3)
C57	0.087 (5)	0.054 (5)	0.041 (4)	0.036 (4)	−0.037 (4)	−0.027 (3)
C58	0.094 (7)	0.109 (8)	0.063 (5)	0.028 (6)	−0.009 (5)	−0.010 (6)
C59	0.122 (7)	0.056 (5)	0.075 (5)	0.022 (5)	−0.054 (5)	−0.010 (4)
C60	0.098 (6)	0.063 (5)	0.075 (6)	0.017 (5)	−0.059 (5)	−0.030 (4)

### Geometric parameters (Å, °)

**Table:**

O1—C11	1.357 (7)	C28—H28B	0.98
O1—C1	1.399 (7)	C28—H28C	0.98
O2—C1	1.203 (8)	C29—C30	1.548 (8)
O3—C12	1.358 (8)	C29—H29A	0.99
O3—H3O	0.84	C29—H29B	0.99
O4—C24	1.352 (8)	C30—H30A	0.98
O4—H4O	0.84	C30—H30B	0.98
O5—C26	1.251 (8)	C30—H30C	0.98
O6—C41	1.369 (7)	C31—C32	1.433 (9)
O6—C31	1.378 (7)	C32—C33	1.348 (9)
O7—C31	1.212 (8)	C32—H32	0.95
O8—C42	1.361 (7)	C33—C40	1.466 (9)
O8—H8O	0.84	C33—C34	1.497 (9)
O9—C54	1.324 (8)	C34—C39	1.379 (8)
O9—H9O	0.84	C34—C35	1.393 (9)
O10—C56	1.230 (9)	C35—C36	1.381 (9)
C1—C2	1.442 (8)	C35—H35	0.95
C2—C3	1.359 (9)	C36—C37	1.406 (9)
C2—H2	0.95	C36—H36	0.95
C3—C10	1.445 (9)	C37—C38	1.362 (10)
C3—C4	1.489 (8)	C37—H37	0.95
C4—C9	1.389 (9)	C38—C39	1.389 (9)
C4—C5	1.393 (9)	C38—H38	0.95
C5—C6	1.390 (9)	C40—C41	1.400 (9)
C5—H5	0.95	C40—C42	1.411 (9)
C6—C7	1.405 (9)	C41—C55	1.421 (10)
C6—H6	0.95	C42—C43	1.368 (9)
C7—C8	1.346 (10)	C39—H39	0.95
C7—H7	0.95	C43—C54	1.411 (9)
C8—C9	1.401 (10)	C43—C44	1.497 (9)
C8—H8	0.95	C44—C45	1.506 (8)
C9—H9	0.95	C44—H44A	0.99
C10—C11	1.406 (8)	C44—H44B	0.99
C10—C12	1.429 (8)	C45—C46	1.332 (9)
C11—C25	1.392 (8)	C45—H45	0.95
C12—C13	1.392 (9)	C46—C48	1.502 (9)
C13—C24	1.369 (9)	C46—C47	1.516 (10)
C13—C14	1.543 (8)	C47—H47A	0.98
C14—C15	1.513 (8)	C47—H47B	0.98
C14—H14A	0.99	C47—H47C	0.98
C14—H14B	0.99	C48—C49	1.540 (9)
C15—C16	1.332 (8)	C48—H48A	0.99
C15—H15	0.95	C48—H48B	0.99
C16—C17	1.503 (9)	C49—C50	1.495 (10)
C16—C18	1.508 (9)	C49—H49A	0.99
C17—H17A	0.98	C49—H49B	0.99
C17—H17B	0.98	C50—C51	1.333 (9)
C17—H17C	0.98	C50—H50	0.95
C18—C19	1.533 (9)	C51—C52	1.496 (11)
C18—H18A	0.99	C51—C53	1.512 (10)
C18—H18B	0.99	C52—H52A	0.98
C19—C20	1.492 (10)	C52—H52B	0.98
C19—H19A	0.99	C52—H52C	0.98
C19—H19B	0.99	C53—H53A	0.98
C20—C21	1.330 (9)	C53—H53B	0.98
C20—H20	0.95	C53—H53C	0.98
C21—C23	1.488 (9)	C54—C55	1.420 (10)
C21—C22	1.512 (9)	C55—C56	1.481 (9)
C22—H22A	0.98	C56—C57	1.531 (7)
C22—H22B	0.98	C57—C59	1.516 (8)
C22—H22C	0.98	C57—C58	1.538 (8)
C23—H23A	0.98	C57—H57	1.00
C23—H23B	0.98	C58—H58C	0.98
C23—H23C	0.98	C58—H58D	0.98
C24—C25	1.433 (8)	C58—H58E	0.98
C25—C26	1.466 (9)	C59—C60	1.535 (8)
C26—C27	1.537 (7)	C59—H59A	0.99
C27—C29	1.495 (8)	C59—H59B	0.99
C27—C28	1.540 (8)	C60—H60A	0.98
C27—H27	1.00	C60—H60B	0.98
C28—H28A	0.98	C60—H60C	0.98
			
C11—O1—C1	124.6 (5)	C29—C30—H30C	109.5
C12—O3—H3O	109.5	H30A—C30—H30C	109.5
C24—O4—H4O	109.5	H30B—C30—H30C	109.5
C41—O6—C31	123.3 (5)	O7—C31—O6	116.3 (6)
C42—O8—H8O	109.5	O7—C31—C32	127.2 (6)
C54—O9—H9O	109.5	O6—C31—C32	116.4 (5)
O2—C1—O1	116.4 (6)	C33—C32—C31	122.0 (6)
O2—C1—C2	127.9 (5)	C33—C32—H32	119.0
O1—C1—C2	115.7 (5)	C31—C32—H32	119.0
C3—C2—C1	121.2 (6)	C32—C33—C40	119.9 (6)
C3—C2—H2	119.4	C32—C33—C34	119.3 (6)
C1—C2—H2	119.4	C40—C33—C34	120.8 (5)
C2—C3—C10	120.1 (6)	C39—C34—C35	119.4 (6)
C2—C3—C4	118.1 (5)	C39—C34—C33	120.8 (5)
C10—C3—C4	121.7 (5)	C35—C34—C33	119.8 (5)
C9—C4—C5	118.4 (6)	C36—C35—C34	119.7 (6)
C9—C4—C3	121.3 (6)	C36—C35—H35	120.1
C5—C4—C3	120.3 (5)	C34—C35—H35	120.1
C6—C5—C4	120.4 (5)	C35—C36—C37	120.4 (6)
C6—C5—H5	119.8	C35—C36—H36	119.8
C4—C5—H5	119.8	C37—C36—H36	119.8
C5—C6—C7	119.9 (6)	C38—C37—C36	119.3 (6)
C5—C6—H6	120.0	C38—C37—H37	120.3
C7—C6—H6	120.0	C36—C37—H37	120.3
C8—C7—C6	119.9 (7)	C37—C38—C39	120.5 (6)
C8—C7—H7	120.1	C37—C38—H38	119.8
C6—C7—H7	120.1	C39—C38—H38	119.8
C7—C8—C9	120.5 (6)	C41—C40—C42	117.0 (6)
C7—C8—H8	119.7	C41—C40—C33	116.8 (6)
C9—C8—H8	119.7	C42—C40—C33	126.2 (6)
C4—C9—C8	120.8 (6)	O6—C41—C40	120.6 (6)
C4—C9—H9	119.6	O6—C41—C55	116.4 (6)
C8—C9—H9	119.6	C40—C41—C55	123.0 (6)
C11—C10—C12	116.0 (6)	O8—C42—C43	115.1 (5)
C11—C10—C3	118.6 (5)	O8—C42—C40	122.1 (6)
C12—C10—C3	125.4 (6)	C43—C42—C40	122.8 (6)
O1—C11—C25	116.7 (5)	C34—C39—C38	120.6 (6)
O1—C11—C10	118.9 (5)	C34—C39—H39	119.7
C25—C11—C10	124.4 (5)	C38—C39—H39	119.7
O3—C12—C13	115.2 (5)	C42—C43—C54	118.5 (6)
O3—C12—C10	123.1 (6)	C42—C43—C44	123.3 (6)
C13—C12—C10	121.7 (6)	C54—C43—C44	118.2 (6)
C24—C13—C12	118.8 (5)	C43—C44—C45	114.5 (5)
C24—C13—C14	121.5 (5)	C43—C44—H44A	108.6
C12—C13—C14	119.7 (6)	C45—C44—H44A	108.6
C15—C14—C13	112.3 (5)	C43—C44—H44B	108.6
C15—C14—H14A	109.2	C45—C44—H44B	108.6
C13—C14—H14A	109.2	H44A—C44—H44B	107.6
C15—C14—H14B	109.2	C46—C45—C44	127.5 (6)
C13—C14—H14B	109.2	C46—C45—H45	116.2
H14A—C14—H14B	107.9	C44—C45—H45	116.2
C16—C15—C14	127.3 (6)	C45—C46—C48	120.0 (6)
C16—C15—H15	116.3	C45—C46—C47	124.6 (6)
C14—C15—H15	116.3	C48—C46—C47	115.3 (6)
C15—C16—C17	124.5 (6)	C46—C47—H47A	109.5
C15—C16—C18	119.9 (6)	C46—C47—H47B	109.5
C17—C16—C18	115.7 (6)	H47A—C47—H47B	109.5
C16—C17—H17A	109.5	C46—C47—H47C	109.5
C16—C17—H17B	109.5	H47A—C47—H47C	109.5
H17A—C17—H17B	109.5	H47B—C47—H47C	109.5
C16—C17—H17C	109.5	C46—C48—C49	114.3 (5)
H17A—C17—H17C	109.5	C46—C48—H48A	108.7
H17B—C17—H17C	109.5	C49—C48—H48A	108.7
C16—C18—C19	113.0 (5)	C46—C48—H48B	108.7
C16—C18—H18A	109.0	C49—C48—H48B	108.7
C19—C18—H18A	109.0	H48A—C48—H48B	107.6
C16—C18—H18B	109.0	C50—C49—C48	111.9 (6)
C19—C18—H18B	109.0	C50—C49—H49A	109.2
H18A—C18—H18B	107.8	C48—C49—H49A	109.2
C20—C19—C18	112.4 (6)	C50—C49—H49B	109.2
C20—C19—H19A	109.1	C48—C49—H49B	109.2
C18—C19—H19A	109.1	H49A—C49—H49B	107.9
C20—C19—H19B	109.1	C51—C50—C49	128.3 (7)
C18—C19—H19B	109.1	C51—C50—H50	115.9
H19A—C19—H19B	107.9	C49—C50—H50	115.9
C21—C20—C19	129.5 (7)	C50—C51—C52	119.9 (7)
C21—C20—H20	115.2	C50—C51—C53	125.6 (7)
C19—C20—H20	115.2	C52—C51—C53	114.5 (6)
C20—C21—C23	124.2 (7)	C51—C52—H52A	109.5
C20—C21—C22	121.2 (6)	C51—C52—H52B	109.5
C23—C21—C22	114.6 (6)	H52A—C52—H52B	109.5
C21—C22—H22A	109.5	C51—C52—H52C	109.5
C21—C22—H22B	109.5	H52A—C52—H52C	109.5
H22A—C22—H22B	109.5	H52B—C52—H52C	109.5
C21—C22—H22C	109.5	C51—C53—H53A	109.5
H22A—C22—H22C	109.5	C51—C53—H53B	109.5
H22B—C22—H22C	109.5	H53A—C53—H53B	109.5
C21—C23—H23A	109.5	C51—C53—H53C	109.5
C21—C23—H23B	109.5	H53A—C53—H53C	109.5
H23A—C23—H23B	109.5	H53B—C53—H53C	109.5
C21—C23—H23C	109.5	O9—C54—C43	116.8 (6)
H23A—C23—H23C	109.5	O9—C54—C55	121.2 (6)
H23B—C23—H23C	109.5	C43—C54—C55	122.0 (6)
O4—C24—C13	116.0 (5)	C54—C55—C41	115.9 (6)
O4—C24—C25	120.7 (6)	C54—C55—C56	118.0 (6)
C13—C24—C25	123.3 (6)	C41—C55—C56	126.0 (6)
C11—C25—C24	115.3 (6)	O10—C56—C55	118.1 (6)
C11—C25—C26	126.8 (5)	O10—C56—C57	118.0 (6)
C24—C25—C26	117.8 (5)	C55—C56—C57	123.8 (7)
O5—C26—C25	119.2 (5)	C59—C57—C56	109.6 (6)
O5—C26—C27	116.2 (6)	C59—C57—C58	120.3 (8)
C25—C26—C27	124.5 (5)	C56—C57—C58	111.1 (6)
C29—C27—C26	108.7 (6)	C59—C57—H57	104.8
C29—C27—C28	117.0 (7)	C56—C57—H57	104.8
C26—C27—C28	111.0 (6)	C58—C57—H57	104.8
C29—C27—H27	106.5	C57—C58—H58C	109.5
C26—C27—H27	106.5	C57—C58—H58D	109.5
C28—C27—H27	106.6	H58C—C58—H58D	109.5
C27—C28—H28A	109.5	C57—C58—H58E	109.5
C27—C28—H28B	109.5	H58C—C58—H58E	109.5
H28A—C28—H28B	109.5	H58D—C58—H58E	109.5
C27—C28—H28C	109.5	C57—C59—C60	110.0 (6)
H28A—C28—H28C	109.5	C57—C59—H59A	109.7
H28B—C28—H28C	109.5	C60—C59—H59A	109.7
C27—C29—C30	110.9 (6)	C57—C59—H59B	109.7
C27—C29—H29A	109.4	C60—C59—H59B	109.7
C30—C29—H29A	109.4	H59A—C59—H59B	108.2
C27—C29—H29B	109.4	C59—C60—H60A	109.5
C30—C29—H29B	109.4	C59—C60—H60B	109.5
H29A—C29—H29B	108.0	H60A—C60—H60B	109.5
C29—C30—H30A	109.5	C59—C60—H60C	109.5
C29—C30—H30B	109.5	H60A—C60—H60C	109.5
H30A—C30—H30B	109.5	H60B—C60—H60C	109.5
			
C11—O1—C1—O2	−173.5 (5)	C41—O6—C31—O7	175.4 (6)
C11—O1—C1—C2	8.8 (8)	C41—O6—C31—C32	−8.2 (8)
O2—C1—C2—C3	176.4 (6)	O7—C31—C32—C33	−180.0 (7)
O1—C1—C2—C3	−6.3 (8)	O6—C31—C32—C33	4.1 (9)
C1—C2—C3—C10	−2.0 (9)	C31—C32—C33—C40	4.6 (9)
C1—C2—C3—C4	174.2 (5)	C31—C32—C33—C34	−174.3 (6)
C2—C3—C4—C9	−112.4 (7)	C32—C33—C34—C39	−65.7 (8)
C10—C3—C4—C9	63.7 (9)	C40—C33—C34—C39	115.5 (7)
C2—C3—C4—C5	68.5 (8)	C32—C33—C34—C35	113.4 (7)
C10—C3—C4—C5	−115.5 (7)	C40—C33—C34—C35	−65.4 (8)
C9—C4—C5—C6	−0.5 (10)	C39—C34—C35—C36	−2.7 (11)
C3—C4—C5—C6	178.7 (6)	C33—C34—C35—C36	178.2 (7)
C4—C5—C6—C7	−0.4 (10)	C34—C35—C36—C37	1.9 (12)
C5—C6—C7—C8	2.2 (11)	C35—C36—C37—C38	−1.0 (11)
C6—C7—C8—C9	−3.0 (12)	C36—C37—C38—C39	0.9 (10)
C5—C4—C9—C8	−0.3 (12)	C32—C33—C40—C41	−9.4 (8)
C3—C4—C9—C8	−179.5 (7)	C34—C33—C40—C41	169.5 (5)
C7—C8—C9—C4	2.1 (13)	C32—C33—C40—C42	170.9 (6)
C2—C3—C10—C11	8.3 (8)	C34—C33—C40—C42	−10.3 (9)
C4—C3—C10—C11	−167.7 (5)	C31—O6—C41—C40	3.2 (9)
C2—C3—C10—C12	−171.2 (6)	C31—O6—C41—C55	−176.8 (5)
C4—C3—C10—C12	12.8 (9)	C42—C40—C41—O6	−174.7 (5)
C1—O1—C11—C25	178.0 (5)	C33—C40—C41—O6	5.6 (8)
C1—O1—C11—C10	−2.7 (8)	C42—C40—C41—C55	5.4 (9)
C12—C10—C11—O1	173.5 (5)	C33—C40—C41—C55	−174.4 (6)
C3—C10—C11—O1	−6.1 (8)	C41—C40—C42—O8	170.0 (5)
C12—C10—C11—C25	−7.2 (8)	C33—C40—C42—O8	−10.3 (9)
C3—C10—C11—C25	173.2 (6)	C41—C40—C42—C43	−9.1 (8)
C11—C10—C12—O3	−170.7 (5)	C33—C40—C42—C43	170.6 (5)
C3—C10—C12—O3	8.8 (9)	C35—C34—C39—C38	2.7 (10)
C11—C10—C12—C13	8.3 (8)	C33—C34—C39—C38	−178.3 (6)
C3—C10—C12—C13	−172.2 (5)	C37—C38—C39—C34	−1.8 (10)
O3—C12—C13—C24	175.3 (5)	O8—C42—C43—C54	−174.7 (5)
C10—C12—C13—C24	−3.8 (8)	C40—C42—C43—C54	4.5 (8)
O3—C12—C13—C14	−6.1 (8)	O8—C42—C43—C44	4.9 (8)
C10—C12—C13—C14	174.9 (5)	C40—C42—C43—C44	−176.0 (5)
C24—C13—C14—C15	−91.3 (7)	C42—C43—C44—C45	−90.6 (7)
C12—C13—C14—C15	90.1 (7)	C54—C43—C44—C45	88.9 (7)
C13—C14—C15—C16	116.9 (7)	C43—C44—C45—C46	−116.6 (7)
C14—C15—C16—C17	−5.9 (10)	C44—C45—C46—C48	−176.9 (6)
C14—C15—C16—C18	174.6 (5)	C44—C45—C46—C47	0.3 (10)
C15—C16—C18—C19	−118.4 (7)	C45—C46—C48—C49	116.9 (7)
C17—C16—C18—C19	62.1 (8)	C47—C46—C48—C49	−60.6 (8)
C16—C18—C19—C20	169.2 (6)	C46—C48—C49—C50	−167.1 (6)
C18—C19—C20—C21	125.9 (8)	C48—C49—C50—C51	−125.9 (8)
C19—C20—C21—C23	−0.9 (11)	C49—C50—C51—C52	−178.5 (7)
C19—C20—C21—C22	179.7 (7)	C49—C50—C51—C53	1.4 (13)
C12—C13—C24—O4	177.2 (5)	C42—C43—C54—O9	−176.8 (6)
C14—C13—C24—O4	−1.4 (8)	C44—C43—C54—O9	3.6 (8)
C12—C13—C24—C25	−2.4 (9)	C42—C43—C54—C55	4.2 (9)
C14—C13—C24—C25	178.9 (5)	C44—C43—C54—C55	−175.3 (6)
O1—C11—C25—C24	−179.1 (5)	O9—C54—C55—C41	173.6 (6)
C10—C11—C25—C24	1.6 (9)	C43—C54—C55—C41	−7.5 (10)
O1—C11—C25—C26	3.3 (10)	O9—C54—C55—C56	−8.7 (10)
C10—C11—C25—C26	−176.0 (7)	C43—C54—C55—C56	170.2 (6)
O4—C24—C25—C11	−176.1 (5)	O6—C41—C55—C54	−177.4 (6)
C13—C24—C25—C11	3.5 (9)	C40—C41—C55—C54	2.5 (9)
O4—C24—C25—C26	1.8 (9)	O6—C41—C55—C56	5.1 (10)
C13—C24—C25—C26	−178.6 (6)	C40—C41—C55—C56	−175.0 (7)
C11—C25—C26—O5	179.9 (7)	C54—C55—C56—O10	12.7 (11)
C24—C25—C26—O5	2.3 (11)	C41—C55—C56—O10	−169.8 (7)
C11—C25—C26—C27	2.7 (12)	C54—C55—C56—C57	−163.7 (7)
C24—C25—C26—C27	−174.9 (6)	C41—C55—C56—C57	13.8 (12)
O5—C26—C27—C29	97.4 (8)	O10—C56—C57—C59	87.9 (9)
C25—C26—C27—C29	−85.3 (9)	C55—C56—C57—C59	−95.7 (9)
O5—C26—C27—C28	−32.5 (10)	O10—C56—C57—C58	−47.5 (12)
C25—C26—C27—C28	144.7 (8)	C55—C56—C57—C58	128.9 (9)
C26—C27—C29—C30	−58.6 (9)	C56—C57—C59—C60	−65.9 (9)
C28—C27—C29—C30	67.9 (10)	C58—C57—C59—C60	64.8 (11)

### Hydrogen-bond geometry (Å, °)

**Table:**

*D*—H···*A*	*D*—H	H···*A*	*D*···*A*	*D*—H···*A*
O4—H4O···O5	0.84	1.70	2.438 (8)	145
O9—H9O···O10	0.84	1.71	2.452 (9)	147
C29—H29B···O5^i^	0.99	2.29	3.115 (10)	140
C32—H32···O2^ii^	0.95	2.43	3.347 (8)	161
C35—H35···O2^iii^	0.95	2.47	3.242 (9)	138
C39—H39···O8^iv^	0.95	2.55	3.434 (8)	155

Symmetry codes: (i) *x*+1, *y*, *z*; (ii) *x*−2, *y*+1, *z*; (iii) *x*−1, *y*+1, *z*; (iv) *x*−1, *y*, *z*.
